# Association of the USPSTF Grade D Recommendation Against Prostate-Specific Antigen Screening With Prostate Cancer–Specific Mortality

**DOI:** 10.1001/jamanetworkopen.2022.11869

**Published:** 2022-05-16

**Authors:** Laura Burgess, Christopher M. Aldrighetti, Anushka Ghosh, Andrzej Niemierko, Fumiko Chino, Melissa J. Huynh, Jason A. Efstathiou, Sophia C. Kamran

**Affiliations:** 1 Division of Radiation Oncology, Department of Radiology, University of Ottawa, Ottawa, Ontario, Canada; 2 Department of Radiation Oncology, Massachusetts General Hospital, Harvard Medical School, Boston; 3 Department of Radiation Oncology, Memorial Sloan Kettering Cancer Center, New York, New York; 4 Division of Urology, Department of Surgery, Western University, London, Ontario, Canada

## Abstract

**Importance:**

The 2012 US Preventive Services Task Force (USPSTF) Grade D recommendation against prostate-specific antigen (PSA) screening for all men has been controversial, with data documenting a shift to a higher stage of disease at diagnosis. The association between the Grade D recommendation and prostate cancer–specific mortality (PCSM) among contemporary cohorts, however, is unclear.

**Objective:**

To evaluate PCSM rates between 1999 and 2019, comparing trends in rates before and after the change in the 2012 USPSTF screening guideline to assess its association with PCSM.

**Exposure:**

The 2012 USPSTF Grade D recommendation against PSA screening for all men.

**Design, Setting, and Participants:**

This cross-sectional study used Centers for Disease Control and Prevention Wide-ranging Online Data for Epidemiologic Research maintained by the National Center for Health Statistics to collect data on cause of death for all individuals who died of prostate cancer in the US from 1999 to 2019. Analysis was performed from January to August 2021.

**Main Outcomes and Measures:**

Trends in PCSM rates were calculated from 1999 to 2012 and from 2014 to 2019, with a washout year of 2013, using linear regression, with year and binary indicator of pre-2013 and post-2013 status as interaction terms. Trends were further analyzed by age, race and ethnicity, urbanization category, and US Census region. Other measures included diagnosis of localized or metastatic prostate cancer and overall cancer mortality.

**Results:**

A total of 618 095 patients died of prostate cancer in the US from 1999 to 2019. Age-adjusted PCSM decreased linearly at a rate of −0.273 per 100 000 population per year from 1999 to 2012 and stalled at a rate of −0.009 per 100 000 per year from 2014 to 2019 (*P* < .001). This finding was significant among men aged 60 years or older, especially among men aged 60 to 69 years, men aged 80 years or older, and among Black men. Men aged 60 to 64 years had a decreasing, age-adjusted PCSM rate of –0.0088 per 100 000 population per year prior to 2013 followed by an increasing rate of 0.0014 per 100 000 per year. Men aged 65 to 69 years had a decreasing, age-adjusted PCSM rate of –0.024 per 100 000 population per year prior to 2013 followed by an increasing rate of 0.0011 per 100 000 population per year. Men aged 80 years or older had the largest absolute difference between rates before and after 2013 compared with all other age groups, with a difference of 0.06 for men aged 80 to 84 years and 0.07 for men 85 aged years or older. Black men had a decreasing, age-adjusted PCSM rate of –0.700 per 100 000 population per year prior to 2013 followed by a flattened rate of –0.091 per 100 000 population per year. Changes were observed across races and ethnicities, urbanization categories, and US Census regions and were accompanied by increased diagnoses of metastatic disease, which are inconsistent with mortality trends across all malignant neoplasms.

**Conclusions and Relevance:**

This cross-sectional study using comprehensive PCSM data through 2019 demonstrated decreasing PCSM rates that flattened or increased after the 2012 USPSTF Grade D recommendation, suggesting that decreased PSA screening may be a factor associated with this change. This change was seen across ages, races and ethnicities, urbanization categories, and US Census regions. The updated 2018 USPSTF guideline supporting shared decision-making may reverse these trends in the coming years.

## Key Points

**Question:**

Was the 2012 US Preventive Services Task Force (USPSTF) Grade D recommendation against prostate-specific antigen (PSA) screening for all men associated with prostate cancer–specific mortality (PCSM)?

**Findings:**

This cross-sectional study found statistically significant changes in PCSM rates that coincided with the change in the screening guideline; PCSM rates were decreasing prior to the recommendation and remained steady after the recommendation.

**Meaning:**

This study suggests that the change in the USPSTF PSA screening guideline to a Grade D recommendation against PSA screening for all men may have been associated with the stagnancy of PCSM rates.

## Teaser

This cross-sectional study evaluates prostate cancer–specific mortality rates between 1999 and 2019, comparing trends in rates before and after the change in the 2012 US Preventive Services Task Force prostate-specific antigen screening guideline.

## Introduction

Prostate cancer remains the most common cancer diagnosed for men in the United States, with an estimated 248 530 new cases and 34 130 deaths in 2021.^1^ The US Preventive Services Task Force (USPSTF) provides recommendations regarding the use of prostate-specific antigen (PSA) screening for prostate cancer. More recently, with evidence that the lifetime risk of a prostate cancer diagnosis far outweighed the lifetime risk of prostate cancer death,^2^ as well as concerns of overdiagnosis or overtreatment, the USPSTF recommended against PSA screening for men older than 75 years in 2008^3^ and subsequently transitioned to a Grade D recommendation against PSA screening for all men in 2012 (draft statement released October 2011).^4^

This recommendation against PSA screening has remained controversial. Several studies have demonstrated that, in the years after the Grade D recommendation, there have been decreases in PSA testing, prostate biopsies, and prostate cancer diagnoses.^5,6^ These decreases have been accompanied by a shift to higher-stage disease, including more metastatic prostate cancers at diagnosis and fewer low-grade prostate cancers.^7,8,9,10^

We sought to understand the association between the 2012 USPSTF Grade D recommendation against PSA screening for all men and prostate cancer–specific mortality (PCSM) using contemporary, comprehensive, population-based nationwide data between 1999 and 2019, comparing mortality rates before and after the 2012 change to the screening guidelines. We also evaluated US trends in diagnoses of both localized prostate cancer and metastatic prostate cancer and in mortality from all malignant neoplasms during the same interval to better understand their association with the Grade D PSA screening recommendation.

## Methods

### Data Sources

The Centers for Disease Control and Prevention (CDC) Wide-ranging Online Data for Epidemiologic Research (WONDER), maintained by the National Center for Health Statistics, contains comprehensive, deidentified mortality data of all individuals in the US, including underlying cause of death.^11^ The database includes patient demographic characteristics, including age, sex, race and ethnicity, and location (reflected in US Census region and urbanization category). To compare trends of localized and metastatic prostate cancer diagnoses in this cross-sectional study, we used the North American Association of Central Cancer Registries. This population-based data set provides comprehensive cancer incidence for North America, including stage at diagnosis.^12^ Analysis was limited to data from the US. This study was exempt from human participant research guidelines by the Mass General Brigham institutional review board owing to secondary analysis of publicly available deidentified data. This study followed the Strengthening the Reporting of Observational Studies in Epidemiology (STROBE) reporting guideline for reporting cross-sectional studies.

### Study Design

The CDC WONDER database was queried for individuals who died of prostate cancer between 1999 and 2019. To establish that observations were exclusive to PCSM, we evaluated mortality data for all cancers among men, as well as among both men and women. Cause of death was based on the *International Statistical Classification of Diseases and Related Health Problems, Tenth Revision* code (prostate cancer code C61). Age-adjusted rates of death were obtained, along with demographic characteristics, including sex, age, race and ethnicity, urbanization category, and US Census region (as per CDC WONDER). Age-adjusted rates per 100 000 population were calculated from the crude rate (count/population × 100 000) and then weighted by the proportion of the persons in the corresponding age groups to the standard population (2000 US standard population).

Age at time of death was reported in 5-year increments starting from 50 years of age. The 2013 National Center for Health Statistics urbanization categories and US Census regions are described in eTables 1 and 2 in the Supplement. Race categories were mutually exclusive and defined as White men and Black (or African American) men. American Indian or Alaska Native men and Asian or Other Pacific Islander men were excluded owing to a paucity of data. Ethnicity was defined as Hispanic (or Latino) men and non-Hispanic (or Latino) men. The North American Association of Central Cancer Registries were used to assess the age-adjusted incidences of localized and metastatic prostate cancers at the time of diagnosis per 100 000 men from 1999 to 2017 (based on data availability).

### Statistical Analysis

Analysis was performed from January to August 2021. Age-adjusted rates of death per 100 000 population were obtained from the CDC WONDER database from 1999 to 2019. Trends in PCSM rates and overall cancer mortality rates were estimated from 1999 to 2012 and from 2014 to 2019, with a washout year of 2013 (consistent with prior analyses^13,14^), using linear regression, with the year and binary indicator of pre-2013 and post-2013 status as interaction terms to assess for statistically significant changes to these trends. Trends were analyzed by race and ethnicity, urbanization category, and US Census region in 5-year age groups. Rates are reported per 100 000 population per year. All *P* values were from 2-sided tests, and results were deemed statistically significant at *P* < .05. All statistical analyses were performed using Stata, version 16.1 (StataCorp LLC).

## Results

Aggregate data for 618 095 patients who died of prostate cancer in the US from 1999 to 2019 were collected. Of these, 103 479 deaths were among Black men and 502 822 were among White men (eTable 3 in the Supplement). There were 32 054 deaths among Hispanic men and 584 711 deaths among non-Hispanic men. The largest proportion of prostate cancer deaths was seen among men aged 70 years or older (504 415 [81.6%]).

**Figure 1.**
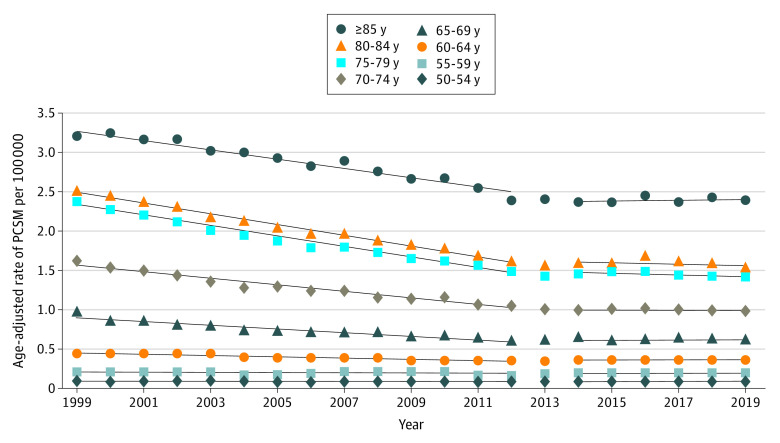
Age-Adjusted Rates of Prostate Cancer–Specific Mortality (PCSM) per 100 000 Population by 5-Year Age Group There was significant (*P* < .001) change in the rate of age-adjusted PCSM after 2013 for men aged 60 years or older. Rates of change were calculated using linear regression, with the interaction term of year and binary indicator of pre-2013 and post-2013 status.

Age-adjusted PCSM decreased linearly at a rate of −0.273 per 100 000 population per year from 1999 to 2012 and subsequently stalled at a rate of −0.009 per 100 000 population per year from 2014 to 2019 (*P* < .001). This finding was significant among men aged 60 years or older, particularly men aged 60 to 69 years and men aged 80 years or older (Figure 1). Men aged 60 to 64 years had a decreasing age-adjusted PCSM rate of −0.0088 per 100 000 population per year prior to 2013 followed by an overall increasing rate of 0.0014 per 100 000 population per year (*P* < .001). Men aged 65 to 69 years had a decreasing age-adjusted PCSM rate of −0.024 per 100 000 population per year prior to 2013 followed by an increasing rate of 0.0011 per 100 000 population per year (*P* < .001) (eTable 4 in the Supplement). Among men aged 80 years or older, the absolute difference between rates before and after 2013 was the largest compared with all other age groups (0.06 for those aged 80 to 84 years and 0.07 for those aged 85 years or older; eTable 4 in the Supplement).

### PCSM Trends by Race

**Figure 2.**
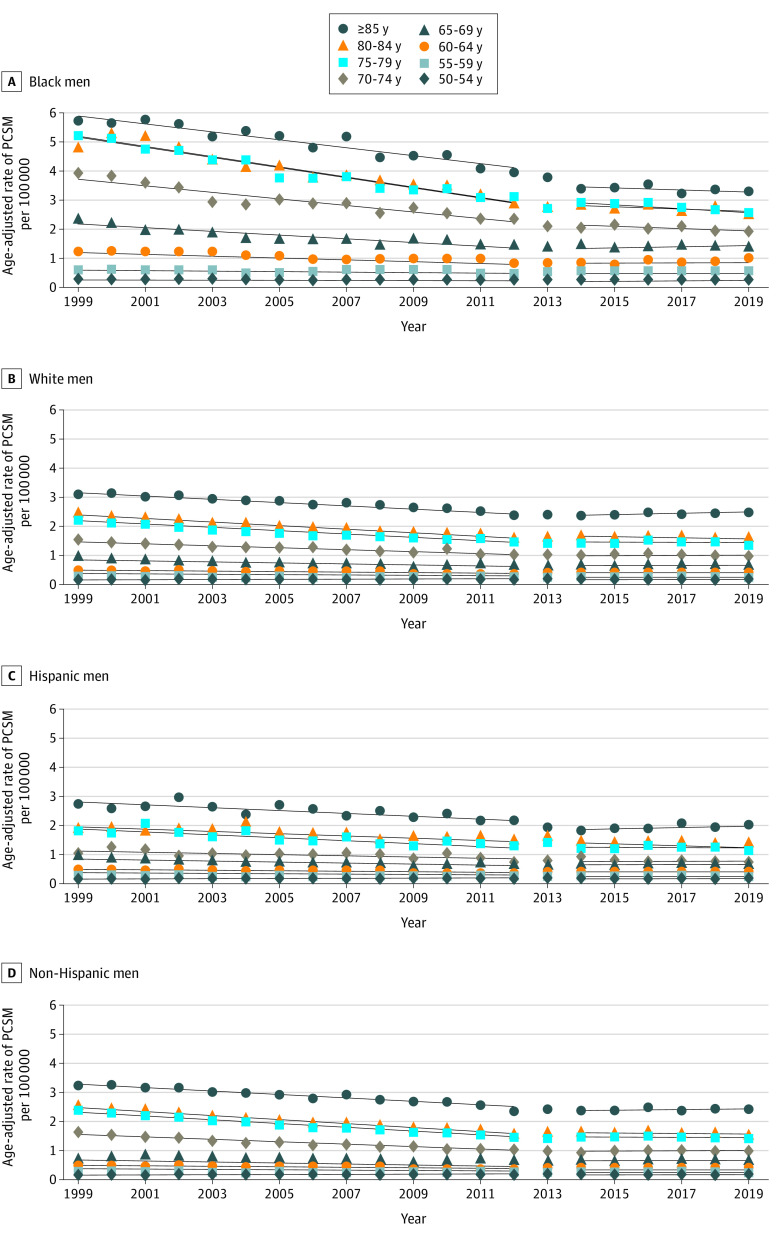
Age-Adjusted Rates of Prostate Cancer–Specific Mortality (PCSM) per 100 000 Population by 5-Year Age Group by Race and Ethnicity A, Among Black men, there was a significant flattening or an increase (*P* < .05) in the age-adjusted rate of PCSM after 2013 for almost all age groups; 50 to 54 years and 85 years or older were the only exceptions. B, White men aged 60 years or older experienced significant (*P* < .001) flattening in the age-adjusted rate of PCSM after 2013, although to a lesser degree compared with Black men. C, Among Hispanic men, there was no significant change observed in age-adjusted PCSM rates before and after 2013. D, Among non-Hispanic men, those aged 60 years or older experienced significant (*P* < .001) flattening in the age-adjusted rate of PCSM after 2012.

When examining PCSM by race, these trends apply to both Black and White men (Figure 2A and B). Among Black men, the age-adjusted PCSM rate decreased linearly at −0.700 per 100 000 population per year from 1999 to 2012 and subsequently flattened at a rate of −0.091 per 100 000 population per year from 2014 to 2019 (*P* < .001). There was significant change in age-adjusted PCSM from 1999 to 2012 and from 2014 to 2019 for Black men aged 55 to 84 years, particularly for men aged 70 years or older, with an absolute difference in age-adjusted PCSM rates greater than 0.1 after 2013 for older men (eTable 5A in the Supplement).

Among White men, the age-adjusted PCSM rate decreased linearly at −0.238 per 100 000 population per year from 1999 to 2012 and subsequently increased at a rate of 0.006 per 100 000 per year from 2014 to 2019 (*P* < .001). There was a significant change in age-adjusted PCSM rates between 1999 to 2012 and 2014 to 2019 for those aged 60 years or older, with absolute differences between PCSM rates ranging between 0.01 and 0.07 (*P* < .001).

### PCSM Trends by Ethnicity

There were no significant changes in PCSM with the change in screening guideline for Hispanic men, but the change was significant among non-Hispanic men aged 60 years or older (Figure 2C and D). Among non-Hispanic men, the age-adjusted rate of PCSM decreased linearly by −0.271 per 100 000 population per year from 1999 to 2012 and subsequently flattened to an annual rate of −0.003 per 100 000 per year from 2014 to 2019 (*P* < .001). There was significant change in the age-adjusted PCSM rate (either stagnated or increased in 2014-2019 vs 1999-2012) for all men aged 60 to 85 years or older, with age-adjusted PCSM rates ranging from –0.0086 to –0.068 per 100 000 population per year from 1999 to 2012, which then stagnated or increased in 2014 to 2019, with age-adjusted PCSM rates ranging from –0.0001 to 0.008 per 100 000 per year (*P* < .001) (eTable 5B in the Supplement).

For Hispanic men, the age-adjusted rate of PCSM decreased linearly by −0.169 per 100 000 population per year from 1999 to 2012 and subsequently stabilized to an annual rate of −0.046 per 100 000 population per year from 2014 to 2019 (*P* = .12). None of the age groups experienced significant changes between 1999 to 2012 and 2014 to 2019.

### PCSM Trends Across Urbanization Categories and Census Regions

These trends were also seen across urbanization categories and US Census regions (eFigure 1 in the Supplement). Within all urbanization categories, the rate of age-adjusted PCSM decreased annually (range, −0.3 to −0.25 per 100 000 population per year) from 1999 to 2012 and then stabilized (range, −0.0036 to 0.024 per 100 000 population per year) from 2014 to 2019 (*P* < .001) (eTable 6A in the Supplement). Similarly, across US Census regions, the rate of age-adjusted PCSM decreased annually (range, −0.3 to −0.22 per 100 000 population per year) from 1999 to 2012 and then flattened out (range, −0.074 to 0.0092 per 100 000 population per year) from 2014 to 2019 (*P* < .001) (eTable 6B in the Supplement).

### Localized or Metastatic Prostate Cancer Diagnoses

**Figure 3.**
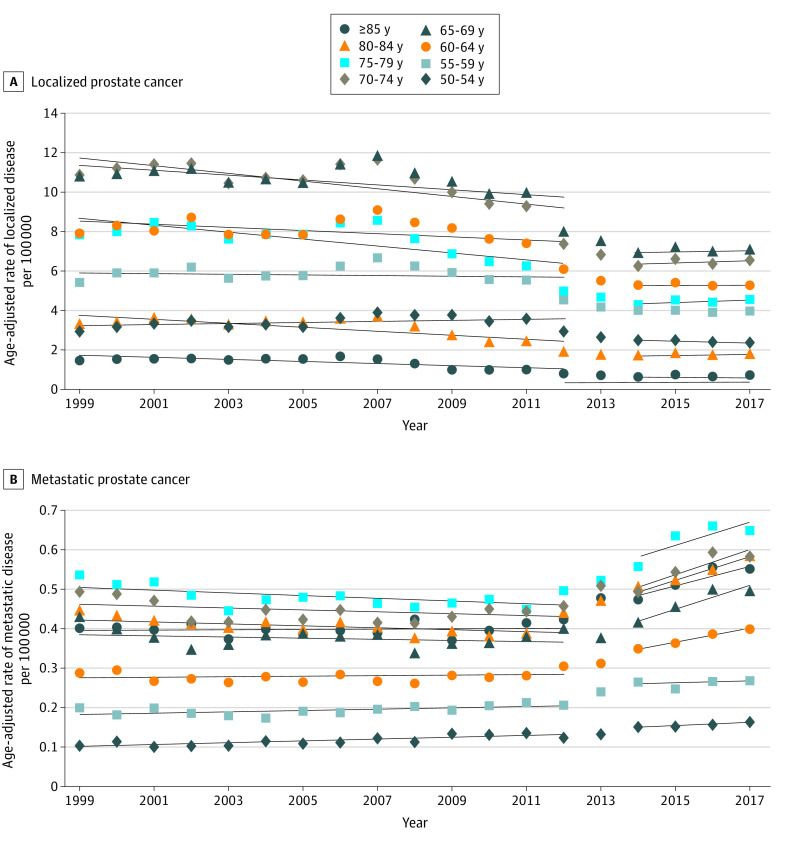
Age-Adjusted Rates of Localized and Metastatic Prostate Cancer Diagnoses Over Time A, Age-adjusted rate of diagnosis of localized prostate cancer per 100 000 population showed a relative increase (*P* ≤ .04) among men aged 70 to 85 years or older. B, Age-adjusted rate of diagnosis of metastatic prostate cancer per 100 000 population significantly (*P* ≤ .03) increased after 2013 among all age groups.

There was an increase in the diagnoses of metastatic prostate cancer that coincided with this change in PCSM that was not observed for localized prostate cancer (Figure 3A and B). Prior to 2013, the rate of age-adjusted incidence of localized prostate cancer decreased. This rate generally flattened after 2013 but was increasing for most age groups. Meanwhile, the age-adjusted incidence of metastatic prostate cancer increased significantly between 1999 to 2012 and 2014 to 2017, particularly among men aged 60 years or older (*P* < .001). Rates of change in age-adjusted diagnosis of metastatic prostate cancer between 1999 and 2012 ranged from –0.004 to 0.001 per 100 000 population per year. This outcome increased to rates of change in age-adjusted diagnosis of metastatic prostate cancer between 2014 and 2017 ranging from 0.02 to 0.03 per 100 000 per year. Men aged 60 to 64 years of age had essentially flat rates of 0.00047 per 100 000 population per year from 1999 to 2012, which increased to 0.017 per 100 000 population per year from 2014 to 2017 (*P* < .001) (eTable 7 in the Supplement). This trend for metastatic prostate cancer was observed across races and ethnicities (eFigure 2A-C in the Supplement).

### Trends in Overall Cancer Mortality

**Figure 4.**
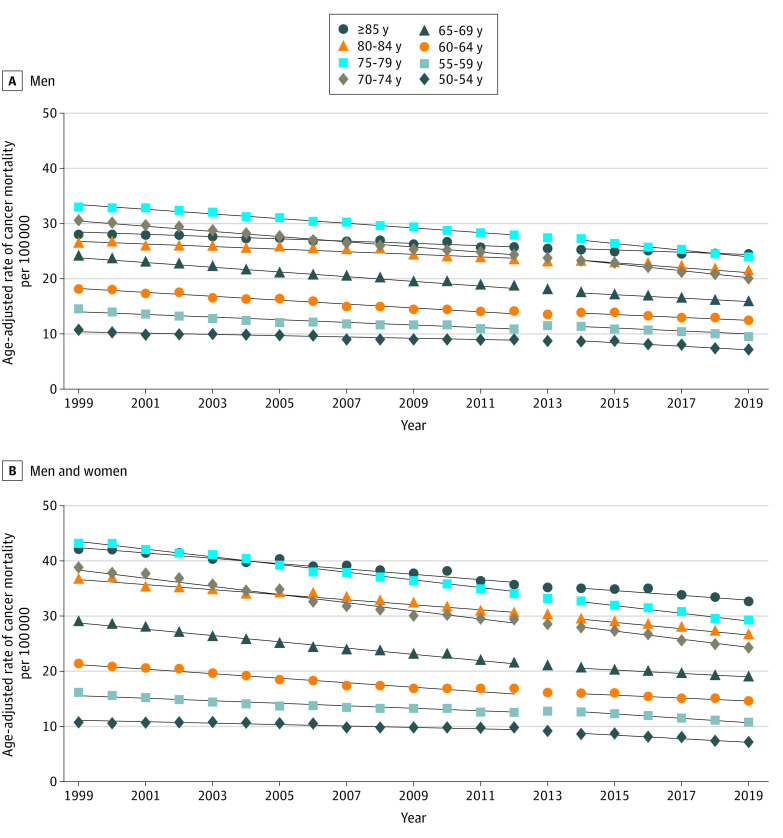
Age-Adjusted Rates of Overall Cancer Mortality per 100 000 Population A, For men, overall cancer mortality decreased steadily from 1999 to 2019. Within many age groups, including men aged 50 to 59 years, the rate of age-adjusted cancer mortality significantly decreased between 1999 to 2012 and 2014 to 2019, the opposite trend to prostate cancer–specific mortality. B, For men and women, overall cancer mortality similarly decreased from 1999 to 2019. Age-adjusted overall cancer mortality decreased significantly between 1999 to 2012 and 2014 to 2019 for all age groups with the exception of individuals aged 60 to 69 years, for whom the rate subsequently flattened after 2013.

The changes seen in prostate cancer mortality were not observed across other cancers. Among men, overall cancer mortality (excluding prostate cancer mortality) decreased steadily from 1999 to 2019. Within many age groups, including men aged 50 to 59 years, the rate of age-adjusted cancer mortality significantly decreased between 1999 to 2012 and 2014 to 2019, the opposite trend to PCSM (Figure 4A). For men aged 60 to 69 years, the rate of age-adjusted overall cancer mortality was lower from 1999 to 2012 (−0.41 per 100 000 population per years for those aged 60-64 years and −0.58 per 100 000 per year for those aged 65-69 years) and increased slightly (although still decreasing on an annual basis) from 2014 to 2019 (−0.3 per 100 000 population per year for those aged 60-64 years and −0.33 per 100 000 population per year for those aged 65-69 years) (*P* < .001) (eTable 8 in the Supplement). For men and women, the age-adjusted overall cancer mortality rate decreased steadily from 1999 to 2019 (Figure 4B). The rate of decrease in age-adjusted overall cancer mortality decreased significantly from 50 to 84 years of age between 1999 to 2012 and 2014 to 2019 except for those aged 60 to 69 years, whereas the age-adjusted overall mortality rate continued to decrease but at a flatter rate from 2014 to 2019.

## Discussion

To our knowledge, this is the first study to report on PCSM rates in response to the USPSTF Grade D recommendation against PSA screening for all men using comprehensive, contemporary population-based data over 20 years (1999-2019), along with comparison with rates of localized and metastatic prostate cancer diagnoses. We have illustrated that significant changes in PCSM rates occurred across age groups, races and ethnicities, and regions, coinciding with the 2012 recommendation against PSA screening. Prior to this recommendation, the PCSM rate was decreasing annually, but this decrease stagnated or increased with the change in the screening guideline. This change also coincided with lower rates of localized prostate cancer diagnoses and higher rates of metastatic prostate cancer diagnoses, without such changes observed in overall cancer mortality.

The benefit of PSA screening has been contested over the past 2 decades. Concerns regarding overdiagnosis or overtreatment,^15,16^ together with 2 major trials,^17,18^ contributed to the 2012 USPSTF Grade D recommendation. The Prostate, Lung, Colorectal and Ovarian Cancer Screening Trial demonstrated no mortality benefit for annual PSA screening after 13 years of follow-up.^17^ However, this trial was intrinsically flawed, with nearly 90% controls undergoing PSA screening^19^ and no mandated biopsy or treatment for an elevated PSA level. Conversely, the European Randomized Screening for Prostate Cancer trial demonstrated a benefit in PCSM associated with PSA screening but also warned of a significant risk of overdiagnosis.^18^ Initial results after a median follow-up of 9 years found that the number needed to screen was 1410 and the number needed to treat was 48 to prevent 1 prostate cancer death. Longer follow-up, however, at 13 and 16 years, demonstrated greater effectiveness of PSA screening, with a lower number needed to screen (n = 570) and a lower number needed to treat (n = 18) to prevent 1 death.^20,21^ This finding, along with evidence demonstrating increased metastatic cancer diagnoses and aggressive prostate cancer diagnoses, as well as evidence of increased use of active surveillance for men with low-risk prostate cancer, prompted the 2018 Grade C recommendation supporting shared decision-making for PSA screening among men 55 to 69 years of age.^22^

The National Health Interview Survey, which collected information on PSA testing among 19 690 men aged 50 years or older in the US in 2010, 2013, and 2015, demonstrated that routine PSA testing decreased from 2010 to 2013 but may have stagnated from 2013 to 2015,^23^ illustrating that PSA testing decreased with the USPSTF recommendations against PSA testing.

Prior studies investigating PSA screening, aggressive disease diagnoses, and prostate cancer deaths after the 2012 screening recommendation and using alternative databases support our findings. Using the Surveillance, Epidemiology, and End Results (SEER) database, Butler et al^24^ demonstrated that the 2012 recommendation was associated with migration toward more aggressive disease. From 2010 to 2015, there was a decrease in the incidence of localized prostate cancer and an increase in the incidence of metastatic prostate cancer among men aged 50 years or older.^24^ Thus, we would expect that changes to PSA screening recommendations may have been associated with the higher rates of metastatic disease that we described herein. Increased use of advanced imaging modalities (eg, prostate-specific membrane antigen positron emission tomography)^25,26^ has also led to upstaging of patients’ disease and higher numbers of patients presenting with upfront metastatic disease.

Sharma et al^27^ investigated the association between PSA screening and the incidence of metastatic prostate cancer using state-level data from the North American Association of Central Cancer Registries from 1999 to 2017 and the Behavioral Risk Factor Surveillance System from 2001 to 2018. They demonstrated that, while PSA screening and the age-adjusted incidence of diagnosis of metastatic prostate cancer in men 40 years or older varied between states, those states with greater decreases in PSA screening had increased incidence of metastatic prostate cancer diagnoses. Other studies have also shown an association between the USPSTF Grade D recommendation and higher-stage disease or higher-grade disease,^8,9,10,28,29^ and several studies have identified sociodemographic variations in the decrease in PSA screening.^30,31^

Kim et al^13^ used SEER registries across the US to assess disparities in prostate cancer–specific survival based on race among White and Black men after the 2012 Grade D recommendation. They assessed men from 2010 to 2016 (similarly using a washout year of 2013) and found a decrease in prostate cancer–specific survival among White men, but not Black men, after the change in recommendation.^13^ Our study, which evaluates PCSM, found a relative increase after the 2012 recommendation in the rate of age-adjusted PCSM among both White and Black men aged 60 years or older. This increase was most striking among Black men, essentially because mortality from prostate cancer was decreasing dramatically prior to the change in screening guidelines. The absolute difference in age-adjusted PCSM rates before and after 2013 across age groups was 0.609 for Black men and 0.244 for White men (*P* < .001). We know that Black men are more likely to receive a diagnosis of prostate cancer, present with higher-stage disease, and have a greater chance of dying from prostate cancer than White men,^32^ but longer follow-up may allow us to understand whether the 2012 recommendation has worsened disparities among Black men with prostate cancer. Differences between our study and the study by Kim et al^13^ could be due to the inclusion of data from 1999 to 2019, comprising a more comprehensive data set.

The significant difference in the rate of change in PCSM observed in our study was seen across all men aged 60 years or older, but it was especially pronounced among those aged 60 to 69 years and those aged 80 years or older. We hypothesize that this difference may be secondary to various changes to the USPSTF recommendations; in 2008, a Grade D recommendation against PSA screening for men aged 75 years or older was issued,^3^ followed by the recommendation against PSA screening for all men in 2012.^4^ This occurrence may explain why PCSM rates flattened or increased from 2014 to 2019 in comparison with pre-2013 rates, with the largest absolute difference in rates among men older than 80 years. The earlier 2008 USPSTF recommendation may have allowed enough time to see a more pronounced association with mortality rates among men in this age group. This trend was again most dramatic for Black men aged 75 years or older, with a noted absolute difference between rates greater than 0.1. Longer follow-up is needed to evaluate whether these trends persist as well as whether PCSM rates increase among younger men after more time, as we suspect. Our observations highlight the need to further investigate the finding of increasing PCSM among older Black men and to assess all associated variables so that physicians can continue to combat and prevent worsening prostate cancer disparities among Black men.

### Limitations and Strengths

This study has some limitations, including those of the CDC WONDER population-based database. Data on disease stage at diagnosis, at time of death, and cancer treatment course for those who died of prostate cancer are not captured. Information about treatment patterns is also not captured. In addition, for a patient’s data to be included, the patient’s death must be reported to the CDC. Among those reported, there may be missing data or inaccuracies. Moreover, the identified cause of death relies on the discretion of the health care professional.^33^ Similar limitations apply to the North American Association of Central Cancer Registries database. However, our study is strengthened by including 2 decades of contemporary PCSM data using the most complete data set of deaths available in the US, spanning both before and after the 2012 USPSTF Grade D recommendation.

Our study had a short follow-up after the 2012 recommendation, particularly considering the long natural history of prostate cancer. Our follow-up period, however, is longer than those found in other similar publications,^13,24,34^ which supports the need for continued follow-up to support our findings. Similarly, the single year as a washout period is short compared with the natural history of prostate cancer, but it is consistent with previously published, population-based data analyses.^13,14^

In addition, there may be a limitation in the use of 2013 as the washout year; the 2012 Grade D USPSTF recommendation was presented in draft form publicly in October 2011, but it can be assumed that practices may not have fully adopted this guideline prior to the official recommendation by the USPSTF; however, the use of 2013 is consistent with prior prostate cancer–specific survival studies.^13^

Last, while previous data demonstrated a strong association between reduced PSA screening and the Grade D USPSTF recommendation,^23,24,27^ we cannot state that this association has directly led to increased PCSM. Patients are not matched within a given database, and we lack information about treatment patterns and disease course. We cannot assume causation, and there are multiple other factors at play. However, we demonstrated that metastatic prostate cancer diagnoses increased and that generally decreasing PCSM rates stagnated after 2013; reduced PSA screening may have been associated with this finding. The findings presented herein show that trends in PCSM and reduced PSA screening are correlated and can lead to the inference that reduced PSA screening was associated with increased PCSM.

## Conclusions

To our knowledge, the present study is the first to evaluate the potential association of the 2012 USPSTF Grade D PSA screening guidelines with PCSM using a comprehensive, contemporary, population-based cohort over 20 years. These findings suggest that the change in the USPSTF PSA screening guideline may have been associated with the observed stagnancy of PCSM rates between 2014 and 2019. The updated 2018 USPSTF guideline supporting shared decision-making may reverse these trends in the coming years. Ongoing discussion surrounding appropriate PSA screening is essential.

## Notes

Supplement. **eTable 1.** 2013 US County and County Equivalent Urbanization **eTable 2.** Census Region and Associated States **eTable 3.** Demographics of Prostate Cancer–Specific Deaths in the United States, 1999-2019 **eTable 4.** Rate of Change in Age-Adjusted PCSM per 100,000 Population by Age **eTable 5A.** Rate of Change in Age-Adjusted PCSM per 100,000 Population by Race **eTable 5B.** Rate of Change in Age-Adjusted PCSM per 100,000 Population by Ethnicity **eTable 6A.** Rate of Change in Age-Adjusted PCSM per 100,000 Population by Urbanization **eTable 6B.** Rate of Change in Age-Adjusted PCSM per 100,000 Population by Census Region **eTable 7.** Rate of Change in Diagnosis of Localized and Metastatic Prostate Cancer **eTable 8.** Rate of Change in Age-Adjusted Overall Cancer Mortality for Men and Women and for Men Alone **eFigure 1.** Age-Adjusted Rate of Prostate Cancer–Specific Mortality (PCSM) per 100,000 by Region **eFigure 2.** Age-Adjusted Rate of Metastatic Prostate Cancer Diagnoses Over Time by Race and Ethnicity
