# Supplementary material for: Association of the USPSTF Grade D Recommendation Against Prostate-Specific Antigen Screening With Prostate Cancer–Specific Mortality
Source: JAMA Netw Open. 2022 May 16;5(5):e2211869. doi: 10.1001/jamanetworkopen.2022.11869 (PMC9112070; doi:10.1001/jamanetworkopen.2022.11869)
Supplement: Supplement. — eTable 1. 2013 US County and County Equivalent Urbanization eTable 2. Census Region and Associated States eTable 3. Demographics of Prostate Cancer–Specific Deaths in the United States, 1999-2019 eTable 4. Rate of Change in Age-Adjusted PCSM per 100,000 Population by Age eTable 5A. Rate of Change in Age-Adjusted PCSM per 100,000 Population by Race eTable 5B. Rate of Change in Age-Adjusted PCSM per 100,000 Population by Ethnicity eTable 6A. Rate of Change in Age-Adjusted PCSM per 100,000 Population by Urbanization eTable 6B. Rate of Change in Age-Adjusted PCSM per 100,000 Population by Census Region eTable 7. Rate of Change in Diagnosis of Localized and Metastatic Prostate Cancer eTable 8. Rate of Change in Age-Adjusted Overall Cancer Mortality for Men and Women and for Men Alone eFigure 1. Age-Adjusted Rate of Prostate Cancer–Specific Mortality (PCSM) per 100,000 by Region eFigure 2. Age-Adjusted Rate of Metastatic Prostate Cancer Diagnoses Over Time by Race and Ethnicity [file jamanetwopen-e2211869-s001.pdf]

Supplementary Online Content

Burgess L, Aldrighetti CM, Ghosh A, et al. Association of the USPSTF Grade D recommendation against prostate-specific antigen screening with prostate cancer–specific mortality. *JAMA Netw Open.* 2022;5(5):e2211869. doi:10.1001/jamanetworkopen.2022.11869

eTable 1. 2013 US County and County Equivalent Urbanization
eTable 2. Census Region and Associated States
eTable 3. Demographics of Prostate Cancer–Specific Deaths in the United States, 1999-2019
eTable 4. Rate of Change in Age-Adjusted PCSM per 100,000 Population by Age
eTable 5A. Rate of Change in Age-Adjusted PCSM per 100,000 Population by Race
eTable 5B. Rate of Change in Age-Adjusted PCSM per 100,000 Population by Ethnicity
eTable 6A. Rate of Change in Age-Adjusted PCSM per 100,000 Population by Urbanization
eTable 6B. Rate of Change in Age-Adjusted PCSM per 100,000 Population by Census Region
eTable 7. Rate of Change in Diagnosis of Localized and Metastatic Prostate Cancer
eTable 8. Rate of Change in Age-Adjusted Overall Cancer Mortality for Men and Women and for Men Alone
eFigure 1. Age-Adjusted Rate of Prostate Cancer–Specific Mortality (PCSM) per 100,000 by Region
eFigure 2. Age-Adjusted Rate of Metastatic Prostate Cancer Diagnoses Over Time by Race and Ethnicity

This supplementary material has been provided by the authors to give readers additional information about their work.

© 2022 Burgess L et al. *JAMA Network Open.*

eTable 1. 2013 US County and County Equivalent Urbanization: Classification as Either Metropolitan or Non-metropolitan and Subclassified Into Six Urbanization Levels Used for Analysis. Urbanization levels are determined by their metropolitan statistical area (MSA) derived from 2012 post-census estimates

County Classification	Urbanization Level	MSA Population	Number of Counties	Distribution of U.S. Resident Population (%)
Metropolitan	Large Central Metro	≥1,000,000	68	30.5
	Large Fringe Metro	1,000,000	368	24.7
	Medium	250,000 – 999,999	373	20.9
	Small	<250,000	358	9.2
Non-Metropolitan	Micropolitan	10,000 – 49,000	641	8.7
	Non-Core	*	1335	6.1

*MSA population cannot be determined for Non-core and should be considered most rural

© 2022 Burgess L et al. *JAMA Network Open.*

eTable 2. Census Region and Associated States: 50 States and Washington D.C. Are Classified Into Four Regions.

Census Region	Northeast	Midwest	South	West
States	Connecticut<br>Maine<br>Massachusetts<br>New Hampshire<br>New Jersey<br>New York<br>Pennsylvania<br>Rhode Island<br>Vermont	Illinois<br>Indiana<br>Iowa<br>Kansas<br>Michigan<br>Minnesota<br>Missouri<br>Nebraska<br>North Dakota<br>Ohio<br>South Dakota<br>Wisconsin	Alabama<br>Arkansas<br>Delaware<br>District of Columbia<br>Florida<br>Georgia<br>Kentucky<br>Louisiana<br>Maryland<br>Mississippi<br>North Carolina<br>Oklahoma<br>South Carolina<br>Tennessee<br>Texas<br>Virginia<br>West Virginia	Alaska<br>Arizona<br>California<br>Colorado<br>Hawaii<br>Idaho<br>Montana<br>Nevada<br>New Mexico<br>Oregon<br>Utah<br>Washington<br>Wyoming

© 2022 Burgess L et al. *JAMA Network Open.*

eTable 3. Demographics of Prostate Cancer–Specific Deaths in the United States, 1999-2019

**Year**	1999	2000	2001	2002	2003	2004	2005	2006	2007	2008	2009
**Demographic**	31729(5.13)	31078(5.03)	30719(4.97)	30446(4.93)	29554(4.78)	29004(4.69)	28905(4.68)	28372(4.59)	29093(4.71)	28472(4.61)	28088(4.54)
**Age**											
<50	137(0.43)	134(0.43)	131(0.43)	114(0.37)	127(0.43)	130(0.45)	122(0.42)	140(0.49)	118(0.41)	122(0.43)	110(0.39)
50-54	274(0.86)	263(0.86)	313(0.86)	302(0.99)	319(0.86)	332(1.14)	302(1.04)	312(0.49)	334(1.15)	361(1.27)	347(1.24)
55-59	636(2.00)	623(2.00)	647(2.00)	696(2.29)	669(2.00)	688(2.37)	762(2.64)	833(2.49)	784(2.69)	817(2.87)	815(2.90)
60-64	1310(4.13)	1306(4.13)	1260(4.13)	1324(4.35)	1405(4.13)	1356(4.68)	1392(4.82)	1440(4.49)	1487(5.11)	1568(5.51)	1573(5.60)
65-69	2711(8.54)	2466(8.54)	2379(8.54)	2316(7.61)	2288(8.54)	2196(7.57)	2215(7.66)	2209(8.49)	2282(7.84)	2365(8.31)	2305(8.21)
70-74	4605(14.51)	4342(14.51)	4164(14.51)	3984(13.09)	3745(14.51)	3515(12.12)	3549(12.28)	3394(14.49)	3434(11.80)	3280(11.52)	3337(11.88)
75-79	6532(20.59)	6314(20.59)	6119(20.59)	5915(19.43)	5671(20.59)	5444(18.77)	5222(18.07)	4924(20.49)	4984(17.13)	4744(16.66)	4502(16.03)
80-84	6903(21.76)	6757(21.76)	6857(21.76)	6838(22.46)	6613(21.76)	6549(22.58)	6444(22.29)	6233(21.49)	6273(21.56)	5977(20.99)	5818(20.71)
85+	8620(27.17)	8873(27.17)	8849(27.17)	8957(29.42)	8717(27.17)	8792(30.31)	8897(30.78)	8887(27.49)	9397(32.30)	9237(32.44)	9281(33.04)
Age not reported	1(0.00)	0(0.00)	0(0.00)	0(0.00)	0(0.00)	2(0.01)	0(0.00)	0(0.49)	0(0.00)	1(0.00)	0(0.00)
All	31729(100.00)	31078(100.00)	30719(100.00)	30446(100.00)	29554(100.00)	29004(100.00)	28905(100.00)	28372(100.49)	29093(100.00)	28472(100.00)	28088(100.00)
**Race**											
White	26005(81.96)	25340(81.96)	25066(81.96)	24918(81.84)	24232(81.96)	23727(81.81)	23597(81.64)	23202(81.49)	23666(81.35)	23362(82.05)	22777(81.09)
Black	5357(16.88)	5346(16.88)	5265(16.88)	5145(16.90)	4894(16.88)	4816(16.60)	4823(16.69)	4698(16.49)	4908(16.87)	4588(16.11)	4794(17.07)
Other races	367(1.16)	392(1.16)	388(1.16)	383(1.26)	428(1.16)	461(1.59)	485(1.68)	472(1.49)	519(1.78)	522(1.83)	517(1.84)
All	31729(100.00)	31078(100.00)	30719(100.00)	30446(100.00)	29554(100.00)	29004(100.00)	28905(100.00)	28372(100.49)	29093(100.00)	28472(100.00)	28088(100.00)
**Ethnicity**											
Hispanic	1050(3.31)	1033(3.31)	1226(3.31)	1209(3.97)	1219(3.31)	1239(4.27)	1300(4.50)	1373(3.49)	1432(4.92)	1436(5.04)	1436(5.11)
Non-Hispanic	30614(96.49)	29964(96.49)	29407(96.49)	29151(95.75)	28279(96.49)	27723(95.58)	27566(95.37)	26946(96.49)	27633(94.98)	26980(94.76)	26604(94.72)
Unknown	65(0.20)	81(0.20)	86(0.20)	86(0.28)	56(0.20)	42(0.14)	39(0.13)	53(0.49)	28(0.10)	56(0.20)	48(0.17)
All	31729(100.00)	31078(100.00)	30719(100.00)	30446(100.00)	29554(100.00)	29004(100.00)	28905(100.00)	28372(100.49)	29093(100.00)	28472(100.00)	28088(100.00)
**Urbanization**											
Large central metro	8778(27.67)	8820(27.67)	8622(27.67)	8608(28.27)	8333(27.67)	8108(27.95)	8263(28.59)	7851(27.49)	7957(27.35)	7803(27.41)	7870(28.02)
Large Fringe metro	6709(21.14)	6446(21.14)	6609(21.14)	6595(21.66)	6311(21.14)	6195(21.36)	6080(21.03)	6075(21.49)	6486(22.29)	6233(21.89)	6200(22.07)
Medium Metro	6493(20.46)	6391(20.46)	6286(20.46)	6208(20.39)	6107(20.46)	6030(20.79)	6003(20.77)	6063(20.49)	6239(21.45)	6161(21.64)	5868(20.89)
Small Metro	3155(9.94)	3131(9.94)	2975(9.94)	3000(9.85)	3034(9.94)	2917(10.06)	2885(9.98)	2900(9.49)	2774(9.53)	2861(10.05)	2809(10.00)
Micropolitan (Non-Metro)	3549(11.19)	3303(11.19)	3304(11.19)	3124(10.26)	3013(11.19)	3036(10.47)	3054(10.57)	2969(11.49)	3101(10.66)	2890(10.15)	2916(10.38)
Non Core (Non-Metro)	3045(9.60)	2987(9.60)	2923(9.60)	2911(9.56)	2756(9.60)	2718(9.37)	2620(9.06)	2514(9.49)	2536(8.72)	2524(8.86)	2425(8.63)
All	31729(100.00)	31078(100.00)	30719(100.00)	30446(100.00)	29554(100.00)	29004(100.00)	28905(100.00)	28372(100.49)	29093(100.00)	28472(100.00)	28088(100.00)
**Census region**											
Northeast	6426(20.25)	6349(20.25)	6386(20.25)	6183(20.31)	6061(20.25)	5778(19.92)	5613(19.42)	5441(20.49)	5591(19.22)	5464(19.19)	5231(18.62)
Midwest	7681(24.21)	7491(24.21)	7374(24.21)	7175(23.57)	6921(24.21)	6887(23.75)	6708(23.21)	6507(24.49)	6786(23.33)	6596(23.17)	6410(22.82)
South	11574(36.48)	11268(36.48)	11110(36.48)	11000(36.13)	10596(36.48)	10430(35.96)	10475(36.24)	10511(36.49)	10566(36.32)	10270(36.07)	10251(36.50)
West	6048(19.06)	5970(19.06)	5849(19.06)	6088(20.00)	5976(19.06)	5909(20.37)	6109(21.13)	5913(19.49)	6150(21.14)	6142(21.57)	6196(22.06)
All	31729(100.00)	31078(100.00)	30719(100.00)	30446(100.00)	29554(100.00)	29004(100.00)	28905(100.00)	28372(100.49)	29093(100.00)	28472(100.00)	28088(100.00)

© 2022 Burgess L et al. *JAMA Network Open.*

Year	2010	2011	2012	2013	2014	2015	2016	2017	2018	2019	Total
**Demographic**	28561(4.62)	27970(4.53)	27245(4.41)	27682(4.48)	28344(4.59)	28848(4.67)	30370(4.91)	30488(4.93)	31489(5.09)	31638(5.12)	618095 (100)
**Age**											
<50	149(0.52)	109(0.39)	124(0.46)	119(0.43)	109(0.38)	86(0.30)	95(0.31)	86(0.28)	119(0.38)	105(0.33)	2486(0.40)
50-54	367(1.28)	331(1.18)	388(1.42)	354(1.28)	333(1.17)	330(1.14)	325(1.07)	324(1.06)	318(1.01)	309(0.98)	6838(1.11)
55-59	907(3.18)	842(3.01)	850(3.12)	878(3.17)	883(3.12)	889(3.08)	968(3.19)	855(2.80)	932(2.96)	915(2.89)	16889(2.73)
60-64	1648(5.77)	1700(6.08)	1611(5.91)	1692(6.11)	1768(6.24)	1826(6.33)	1925(6.34)	1888(6.19)	1984(6.30)	2007(6.34)	33470(5.42)
65-69	2452(8.59)	2412(8.62)	2481(9.11)	2613(9.44)	2886(10.18)	2814(9.75)	3048(10.04)	3184(10.44)	3174(10.08)	3188(10.08)	53984(8.73)
70-74	3414(11.95)	3270(11.69)	3338(12.25)	3371(12.18)	3504(12.36)	3673(12.73)	3833(12.62)	4096(13.43)	4246(13.48)	4402(13.91)	78496(12.70)
75-79	4435(15.53)	4350(15.55)	4236(15.55)	4187(15.13)	4331(15.28)	4409(15.28)	4641(15.28)	4755(15.60)	4992(15.85)	5054(15.97)	105761(17.11)
80-84	5700(19.96)	5503(19.67)	5145(18.88)	5072(18.32)	5133(18.11)	5202(18.03)	5440(17.91)	5388(17.67)	5469(17.37)	5433(17.17)	124747(20.18)
85+	9488(33.22)	9453(33.80)	9071(33.29)	9395(33.94)	9396(33.15)	9619(33.34)	10095(33.24)	9910(32.50)	10254(32.56)	10223(32.31)	195411(31.62)
Age not reported	1(0.00)	0(0.00)	1(0.00)	1(0.00)	1(0.00)	0(0.00)	0(0.00)	2(0.01)	1(0.00)	2(0.01)	13(0.00)
All	28561(100.00)	27970(100.00)	27245(100)	27682(100.00)	28344(100.00)	28848(100.00)	30370(100.00)	30488(100.00)	31489(100.00)	31638(100.00)	618095(100.00)
**Race**											
White	23172(81.13)	22728(81.26)	22093(81.09)	22550(81.46)	23073(81.40)	23377(81.04)	24555(80.85)	24659(80.88)	25316(80.40)	25407(80.31)	502822(81.35)
Black	4854(17.00)	4658(16.65)	4596(16.87)	4528(16.36)	4613(16.28)	4742(16.44)	5088(16.75)	5065(16.61)	5291(16.80)	5410(17.10)	103479(16.74)
Other races	535(1.87)	584(2.09)	556(2.04)	604(2.18)	658(2.32)	729(2.53)	727(2.39)	764(2.51)	882(2.80)	821(2.59)	11794(1.91)
All	28561(100.00)	27970(100.00)	27245(100.00)	27682(100.00)	28344(100.00)	28848(100.00)	30370(100.00)	30488(100.00)	31489(100.00)	31638(100.00)	618095(100.00)
**Ethnicity**											
Hispanic	1535(5.37)	1576(5.63)	1592(5.84)	1634(5.90)	1707(6.02)	1867(6.47)	1929(6.35)	2011(6.60)	2115(6.72)	2135(6.75)	32054(5.19)
Non-Hispanic	26973(94.44)	26343(94.18)	25596(93.95)	25983(93.86)	26538(93.63)	26908(93.28)	28361(93.38)	28405(93.17)	29297(93.04)	29440(93.05)	584711(94.60)
Unknown	53(0.19)	51(0.18)	57(0.21)	65(0.23)	99(0.35)	73(0.25)	80(0.26)	72(0.24)	77(0.24)	63(0.20)	1330(0.22)
All	28561(100.00)	27970(100.00)	27245(100.00)	27682(100.00)	28344(100.00)	28848(100.00)	30370(100.00)	30488(100.00)	31489(100.00)	31638(100.00)	618095(100.00)
**Urbanization**											
Large central metro	7936(27.79)	7921(28.32)	7607(27.92)	7691(27.78)	8012(28.27)	7931(27.49)	8557(28.18)	8421(27.62)	8743(27.77)	8877(28.06)	172709(27.94)
Large Fringe metro	6283(22.00)	6232(22.28)	6015(22.08)	6318(22.82)	6446(22.74)	6610(22.91)	6912(22.76)	7163(23.49)	7220(22.93)	7373(23.30)	136511(22.09)
Medium Metro	5992(20.98)	5777(20.65)	5747(21.09)	5755(20.79)	6039(21.31)	6149(21.32)	6321(20.81)	6473(21.23)	6735(21.39)	6688(21.14)	129525(20.96)
Small Metro	2906(10.17)	2779(9.94)	2716(9.97)	2761(9.97)	2772(9.78)	2876(9.97)	3146(10.36)	2990(9.81)	3233(10.27)	3142(9.93)	61762(9.99)
Micropolitan (Non-Metro)	2934(10.27)	2921(10.44)	2790(10.24)	2778(10.04)	2776(9.79)	2956(10.25)	2966(9.77)	2997(9.83)	3120(9.91)	3113(9.84)	63610(10.29)
Non Core (Non-Metro)	2510(8.79)	2340(8.37)	2370(8.70)	2379(8.59)	2299(8.11)	2326(8.06)	2468(8.13)	2444(8.02)	2438(7.74)	2445(7.73)	53978(8.73)
All	28561(100.00)	27970(100.00)	27245(100.00)	27682(100.00)	28344(100.00)	28848(100.00)	30370(100.00)	30488(100.00)	31489(100.00)	31638(100.00)	618095(100.00)
**Census region**											
Northeast	5458(19.11)	5318(19.01)	5024(18.44)	5112(18.47)	5207(18.37)	5079(17.61)	5398(17.77)	5439(17.84)	5391(17.12)	5296(16.74)	117245(18.97)
Midwest	6466(22.64)	6318(22.59)	6173(22.66)	6093(22.01)	6334(22.35)	6438(22.32)	6604(21.75)	6713(22.02)	6953(22.08)	6928(21.90)	141556(22.90)
South	10428(36.51)	10171(36.36)	10021(36.78)	10237(36.98)	10323(36.42)	10617(36.80)	11141(36.68)	11357(37.25)	11628(36.93)	11963(37.81)	225937(36.55)
West	6209(21.74)	6163(22.03)	6027(22.12)	6240(22.54)	6480(22.86)	6714(23.27)	7227(23.80)	6979(22.89)	7517(23.87)	7451(23.55)	133357(21.58)
All	28561(100.00)	27970(100.00)	27245(100.00)	27682(100.00)	28344(100.00)	28848(100.00)	30370(100.00)	30488(100.00)	31489(100.00)	31638(100)	618095(100.00)

© 2022 Burgess L et al. *JAMA Network Open.*

eTable 4. Rate of Change in Age-Adjusted PCSM per 100,000 Population by Age

Age Group	Rate of change in age-adjusted PCSM (per 100,000 population) pre-2013	Rate of change in age-adjusted PCSM (per 100,000 population) post-2013	Absolute difference between age-adjusted PCSM rates of change pre-2013 and post-2013*	p-value
50 – 54	+0.000017	+0.00059	0.000573	0.20
55 – 59	-0.0018	+0.00053	0.00233	0.47
60 – 64	-0.0088	+0.0014	0.0103	**<0.001**
65 – 69	-0.024	+0.0011	0.0251	**<0.001**
70 – 74	-0.042	-0.0026	0.0016	**<0.001**
75 – 79	-0.065	-0.011	0.0045	**<0.001**
80 – 84	-0.069	-0.0098	0.0592	**<0.001**
85+	-0.062	+0.0078	0.0698	**<0.001**
All ages	-0.273	-0.009	0.264	**<0.001**

*To evaluate the differences in rates of change in age-adjusted PCSM we subtracted the post-2013 age-adjusted rate of PCSM from the pre-2013 age-adjusted rate of PCSM to understand the magnitude of change in the rate of age-adjusted PCSM between these time periods

© 2022 Burgess L et al. *JAMA Network Open.*

eTable 5A. Rate of Change in Age-Adjusted PCSM per 100,000 Population by Race

Race	Age Group	Rate of change in age-adjusted PCSM (per 100,000 population) pre-2013	Rate of change in age-adjusted PCSM (per 100,000 population) post-2013	Absolute difference between age-adjusted PCSM rates of change pre-2013 and post-2013*	p-value
Black or African American	50 – 54	-0.0032	+0.0088	0.012	0.12
	55 – 59	-0.0098	+0.0056	0.0154	**0.02**
	60 – 64	-0.03	+0.0099	0.0399	**0.001**
	65 – 69	-0.06	+0.025	0.085	**0.001**
	70 – 74	-0.12	-0.028	0.092	**0.007**
	75 – 79	-0.18	-0.06	0.12	**<0.001**
	80 – 84	-0.17	-0.032	0.138	**<0.001**
	85+	-0.14	-0.03	0.11	0.06
	All ages	-0.700	-0.091	0.609	**<0.001**
White	50 – 54	+0.00009	-0.00065	0.00074	0.48
	55 – 59	-0.0014	-0.00017	0.00123	0.84
	60 – 64	-0.0063	-0.00035	0.00595	**<0.001**
	65 – 69	-0.019	-0.0039	0.0151	**<0.001**
	70 – 74	-0.036	+0.000017	0.036017	**<0.001**
	75 – 79	-0.056	-0.0038	0.0522	**<0.001**
	80 – 84	-0.06	-0.0083	0.0517	**<0.001**
	85+	-0.055	+0.014	0.069	**<0.001**
	All ages	-0.238	+0.006	0.244	**<0.001**

*To evaluate the differences in rates of change in age-adjusted PCSM we subtracted the post-2013 age-adjusted rate of PCSM from the pre-2013 age-adjusted rate of PCSM to understand the magnitude of change in the rate of age-adjusted PCSM between these time periods

© 2022 Burgess L et al. *JAMA Network Open.*

eTable 5B. Rate of Change in Age-Adjusted PCSM per 100,000 Population by Ethnicity

Ethnicity	Age Group	Rate of change in age-adjusted PCSM (per 100,000 population) pre-2013	Rate of change in age-adjusted PCSM (per 100,000 population) post-2013	Absolute difference between age-adjusted PCSM rates of change pre-2013 and post-2013*	p-value
Hispanic or Latino	50 – 54	-0.0016	-0.0023	0.0007	0.68
	55 – 59	-0.0026	-0.0002	0.0024	0.61
	60 – 64	-0.0015	-0.0014	0.0001	0.61
	65 – 69	-0.015	-0.0061	0.0089	0.74
	70 – 74	-0.022	-0.019	0.003	0.77
	75 – 79	-0.046	-0.0024	0.0436	0.09
	80 – 84	-0.039	-0.033	0.006	0.72
	85+	-0.052	+0.026	0.078	0.07
	All ages	-0.169	-0.046	0.123	0.12
Non-Hispanic or Latino	50 – 54	+0.00023	+0.0013	0.00107	0.11
	55 – 59	-0.0017	+0.001	0.0027	0.32
	60 – 64	-0.0086	+0.0023	0.0109	**<0.001**
	65 – 69	-0.023	+0.0027	0.0257	**<0.001**
	70 – 74	-0.042	-0.000098	0.041902	**<0.001**
	75 – 79	-0.064	-0.011	0.053	**<0.001**
	80 – 84	-0.068	-0.0071	0.0609	**<0.001**
	85+	-0.062	+0.0084	0.0704	**<0.001**
	All ages	-0.271	-0.003	0.268	**<0.001**

*To evaluate the differences in rates of change in age-adjusted PCSM we subtracted the post-2013 age-adjusted rate of PCSM from the pre-2013 age-adjusted rate of PCSM to understand the magnitude of change in the rate of age-adjusted PCSM between these time periods

© 2022 Burgess L et al. *JAMA Network Open.*

eTable 6A. Rate of Change in Age-Adjusted PCSM per 100,000 Population by Urbanization

Urbanization	Rate of change in age-adjusted PCSM (per 100,000 population) pre-2013	Rate of change in age-adjusted PCSM (per 100,000 population) post-2013	Absolute difference between age-adjusted PCSM rates of change pre-2013 and post-2013*	p-value
Large Central Metro	-0.25	-0.026	0.224	**<0.001**
Large Fringe Metro	-0.26	-0.0012	0.2588	**<0.001**
Medium Metro	-0.27	-0.023	0.247	**<0.001**
Small Metro	-0.28	+0.022	0.302	**<0.001**
Micropolitan Non-Metro	-0.27	+0.024	0.294	**<0.001**
Non-Core Non-Metro	-0.3	-0.036	0.264	**<0.001**
All Categories	-0.27	-0.0086	0.2614	**<0.001**

*To evaluate the differences in rates of change in age-adjusted PCSM we subtracted the post-2013 age-adjusted rate of PCSM from the pre-2013 age-adjusted rate of PCSM to understand the magnitude of change in the rate of age-adjusted PCSM between these time periods

© 2022 Burgess L et al. *JAMA Network Open.*

eTable 6B. Rate of Change in Age-Adjusted PCSM per 100,000 Population by Census Region

Census Region	Rate of change in age-adjusted PCSM (per 100,000 population) pre-2013	Rate of change in age-adjusted PCSM (per 100,000 population) post-2013	Absolute difference between age-adjusted PCSM rates of change pre-2013 and post-2013*	p-value
Northeast	-0.27	-0.074	0.196	**<0.001**
Midwest	-0.28	+0.0092	0.2892	**<0.001**
South	-0.3	-0.0032	0.2968	**<0.001**
West	-0.22	+0.0065	0.2265	**<0.001**
All Regions	-0.27	-0.0086	0.2614	**<0.001**

*To evaluate the differences in rates of change in age-adjusted PCSM we subtracted the post-2013 age-adjusted rate of PCSM from the pre-2013 age-adjusted rate of PCSM to understand the magnitude of change in the rate of age-adjusted PCSM between these time periods

© 2022 Burgess L et al. *JAMA Network Open.*

eTable 7. Rate of Change in Diagnosis of Localized and Metastatic Prostate Cancer

Diagnosis	Age Group	Rate of change in age-adjusted diagnosis of prostate cancer (per 100,000 population) pre-2013	Rate of change in age-adjusted diagnosis of prostate cancer (per 100,000 population) post-2013	Absolute difference between age-adjusted diagnosis of prostate cancer change pre-2013 and post-2013*	p-value
Localized‡	50 – 54	-0.19	-0.046	0.144	0.21
	55 – 59	-0.37	-0.024	0.346	0.07
	60 – 64	-0.54	+0.015	0.555	**0.04**
	65 – 69	-0.64	-0.038	0.602	0.08
	70 – 74	-0.74	+0.054	0.794	**0.04**
	75 – 79	-0.59	+0.067	0.657	**0.03**
	80 – 84	-0.28	+0.016	0.296	**0.01**
	85+	-0.13	-0.0066	0.1234	**0.008**
Metastatic	50 – 54	+0.0024	+0.0044	0.002	**0.03**
	55 – 59	+0.0017	+0.0024	0.0007	**<0.001**
	60 – 64	+0.00047	+0.017	0.01653	**<0.001**
	65 – 69	-0.0016	+0.029	0.0306	**<0.001**
	70 – 74	-0.0024	+0.031	0.0334	**<0.001**
	75 – 79	-0.0038	+0.03	0.0338	**<0.001**
	80 – 84	-0.0025	+0.03	0.0325	**<0.001**
	85+	+0.00064	+0.025	0.02436	**<0.001**

*To evaluate the differences in rates of change in age-adjusted diagnosis of prostate cancer, we subtracted the post-2013 age-adjusted rate of prostate cancer diagnoses from the pre-2013 age-adjusted rate of prostate cancer diagnoses to understand the magnitude of change in the rate of age-adjusted prostate cancer diagnoses between these time periods

‡Localized prostate cancer trends were analyzed from 2008-2012 and from 2014-2017

© 2022 Burgess L et al. *JAMA Network Open.*

eTable 8. Rate of Change in Age-Adjusted Overall Cancer Mortality** for Men and Women and for Men Alone

Sex	Age Group	Rate of change in age-adjusted overall cancer mortality (per 100,000 population) pre-2013	Rate of change in age-adjusted overall cancer mortality (per 100,000 population) post-2013	Absolute difference between age-adjusted overall cancer mortality rates of change pre-2013 and post-2013*	p-value
Men	50 – 54	-0.13	-0.34	0.21	**<0.001**
	55 – 59	-0.23	-0.4	0.17	**0.05**
	60 – 64	-0.41	-0.3	0.11	**<0.001**
	65 – 69	-0.58	-0.33	0.25	**<0.001**
	70 – 74	-0.75	-0.77	0.02	0.09
	75 – 79	-0.71	-0.75	0.04	0.25
	80 – 84	-0.46	-0.56	0.1	**0.04**
	85+	-0.5	-0.46	0.04	0.9
Men and Women	50 – 54	-0.13	-0.28	0.15	**<0.001**
	55 – 59	-0.23	-0.29	0.06	**0.005**
	60 – 64	-0.35	-0.19	0.16	**<0.001**
	65 – 69	-0.43	-0.32	0.11	**0.007**
	70 – 74	-0.5	-0.61	0.11	**0.05**
	75 – 79	-0.41	-0.6	0.19	**<0.001**
	80 – 84	-0.23	-0.39	0.16	**<0.001**
	85+	-0.18	-0.21	0.03	0.33

*To evaluate the differences in rates of change in age-adjusted overall cancer mortality we subtracted the post-2013 age-adjusted rate of overall cancer mortality from the pre-2013 age-adjusted rate of overall cancer mortality to understand the magnitude of change in the rate of age-adjusted overall cancer mortality between these time periods

**Overall cancer mortality rates excluded prostate cancer mortality

© 2022 Burgess L et al. *JAMA Network Open.*

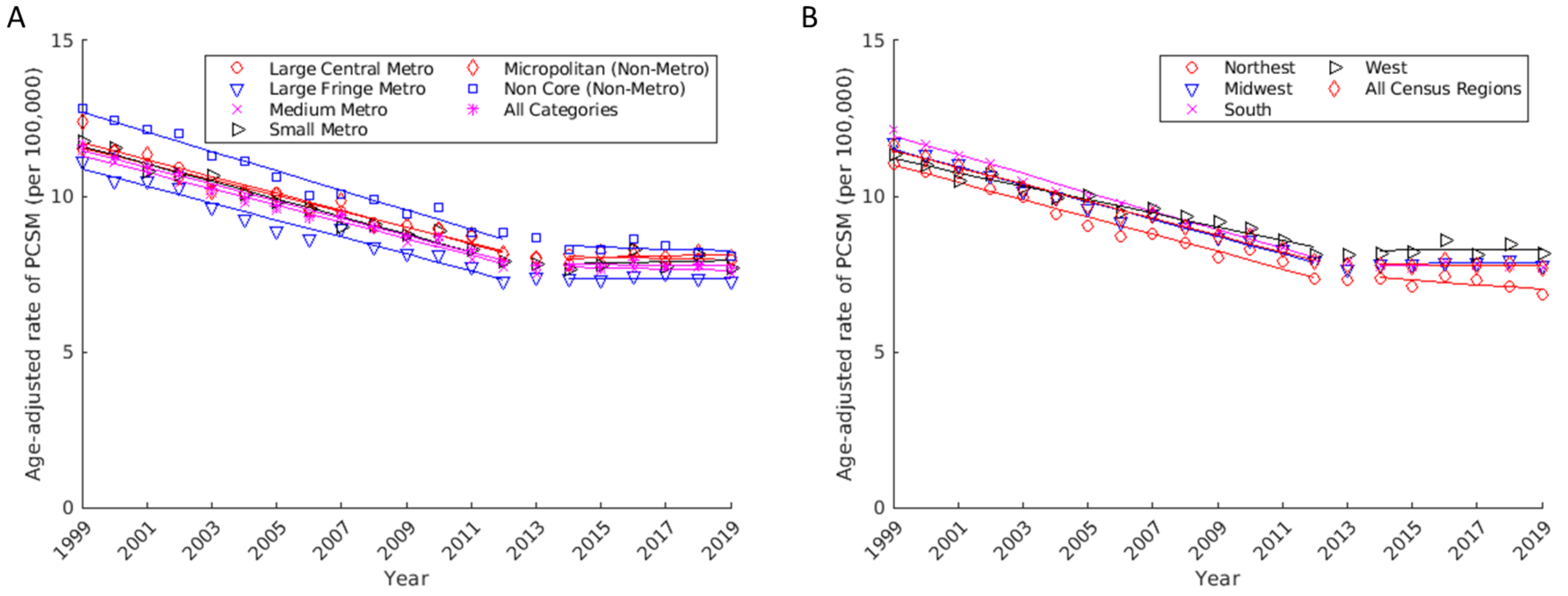

eFigure 1. Age-Adjusted Rate of Prostate Cancer–Specific Mortality (PCSM) per 100,000 by Region

By region, the age-adjusted rate of PCSM was steadily decreasing yearly between 1999-2012 and significantly ($p<0.001$) flattened after 2013 across all (A) urbanization categories and (B) census regions.

© 2022 Burgess L et al. *JAMA Network Open.*

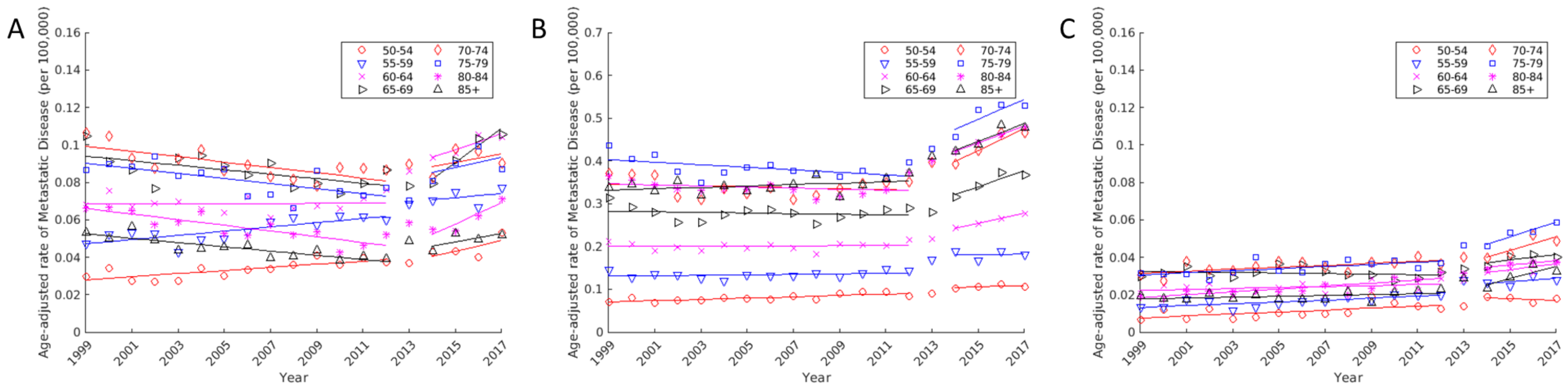

eFigure 2. Age-Adjusted Rate of Metastatic Prostate Cancer Diagnoses Over Time by Race and Ethnicity. (A) In Black men, (B) White men and (C) Hispanic men, there was a significant increase in the age-adjusted rate of diagnosis of metastatic prostate cancer per 100,000 post-2013 in all age groups.

© 2022 Burgess L et al. *JAMA Network Open.*
